# Involvement of post-transcriptional regulation of FOXO1 by HuR in 5-FU-induced apoptosis in breast cancer cells

**DOI:** 10.3892/ol.2013.1352

**Published:** 2013-05-17

**Authors:** YUNBO LI, JINHAI YU, DANHUA DU, SHUANGLIN FU, YE CHEN, FANG YU, PENG GAO

**Affiliations:** 1 Norman Bethune College of Medicine, Jilin University, Changchun, Jilin 130041;; 2 The First Hospital of Jilin University, Changchun, Jilin 130041;; 3 Department of Nutrition and Food Hygiene, Fourth Military Medical University, Xi’an 710032, P.R. China

**Keywords:** FOXO1, HuR, 5-FU, breast cancer, apoptosis

## Abstract

The post-transcriptional control of specific mRNAs is a widespread mechanism of gene regulation, which contributes to numerous biological processes in a number of cell types. The Forkhead box O (FoxO) transcription factor FOXO1 is an important tumor suppressor involved in apoptosis, the cell cycle, DNA damage repair and oxidative stress. Bioinformatic prediction identified that the 3′ untranslated region (UTR) of FOXO1 is enriched with binding motifs for the human ELAV/Hu protein (HuR), indicating that FOXO1 is a potential target of HuR. Luciferase reporter assays demonstrate that HuR specifically regulates FOXO1 expression through AU-rich elements (AREs) within the FOXO1 3′ UTR. Immunoprecipitation studies confirmed that HuR associates with FOXO1 mRNA in MDA-MB-231 breast cancer cells and that HuR upregulates FOXO1 mRNA levels through increased mRNA stability. Using a HuR loss- and gain-of-function approach, we revealed that FOXO1 expression was correspondingly decreased or increased in MDA-MB-231 cells. Functional assays demonstrated that HuR and FOXO1 expression levels were markedly enhanced upon 5-fluorouracil (5-FU) stimulation in MDA-MB-231 cells. Knockdown of HuR apparently abrogated 5-FU-induced apoptosis detected by caspase-3 activities. Furthermore, in HuR knockdown cells, additional overexpression of FOXO1 moderately recovered 5-FU-induced apoptosis, which verified that HuR-modulated apoptosis upon 5-FU treatment was partially mediated by its post-transcriptional regulation of FOXO1. Therefore, modulating FOXO1 expression has been suggested to lead to the development of new therapeutic treatments for certain types of cancer.

## Introduction

Although the majority of gene expression regulation occurs at the time of transcription, translational control of specific mRNAs through the regulation of mRNA stability, localization and translation ability determines the spatial and temporal expression in many cell types (1,2).

The hu antigen R (HuR) is a large, highly conserved RNA-binding protein that is involved in the shuttling of transcripts from the nucleus into the cytoplasm (3), as well as the regulation of mRNA stability and translation (4,5). HuR binds specifically to translational control elements in the target mRNA 3′ untranslated regions (UTRs) known as Nanos response elements (NREs) (4,6). HuR has been implicated in cell growth and differentiation via the regulation of mRNA expression in the cytoplasm (7). In human colorectal carcinoma cells, UV irradiation elevates the rate of p21 mRNA translation in a HuR-dependent manner (8). In the cytoplasm, HuR-containing mRNA complexes cofractionate with polysomes (9). Additionally, the binding of p53 mRNA to polysomes and its increased translation is HuR-mediated (9). Moreover, high cytoplasmic levels of HuR have been associated with a higher tumor grade, increased cyclooxygenase-2 expression and poor survival rates in breast carcinoma (10), suggesting a role for HuR in cancer pathogenesis.

The Forkhead box O (FoxO) transcription factor FOXO1 is emerging as an important tumor suppressor that modulates the expression of genes involved in apoptosis, the cell cycle, DNA damage repair and oxidative stress (11–13). FOXO1 can be regulated by a number of mechanisms. It has been widely accepted that phosphorylation of the three PKB/Akt consensus sites in FOXO1 following incubation with insulin or other serum components, results in a rapid export of FOXO proteins from the nucleus to the cytoplasm (12,14,15), which inhibits the FOXO-stimulated transcription of target genes.

In the current study, we demonstrate that HuR positively regulates FOXO1 expression via the 3′ UTR upon 5-fluorouracil (5-FU) stimulation, which results in enhanced mRNA stability. Our study suggests that in addition to post-translational modification, post-transcriptional mechanisms, including mRNA stability and translation, are critical in the control of FOXO1 expression.

## Materials and methods

### Cell culture

The MDA-MB-231 human breast cancer cell line was grown in Dulbecco’s modified Eagle’s medium supplemented with 10% inactivated fetal bovine serum, 2 mM L-glutamine, 50 U/ml penicillin and 50 *μ*g/ml streptomycin at 37°C in a humidified atmosphere of 5% CO_2_.

### Plasmids and Stealth siRNAs™

HuR and FOXO1 overexpression vectors (pcDNA-flag-HuR and pcDNA-flag-FOXO1, respectively) were generated by cloning PCR-amplified sequences into pcDNA3.0-flag vectors with *Eco*RI and *Bam*HI restriction enzymes. The FOXO1 3′ UTR reporter plasmid (designated as WT) was constructed by cloning PCR-amplified sequences from the 3′ UTR of FOXO1 cDNA into the *Xba*I site of a pGL3 luciferase reporter vector (Promega, Madison, WI, USA). Two sites of the pGL3-FOXO1-3′ UTR seed sequence were deleted (designated as Mutant). The siRNA duplex targeting human HuR is 5′-AAGCCUGUUCAGCAGCAUUGG-3′ (Dharmacon, Inc., Lafayette, CO, USA).

### Luciferase assays

MDA-MB-231 cells were seeded into 24-well plates and transiently transfected with 400 ng of FOXO1 3′ UTR reporter plasmid (WT or Mutant) in combination with increased doses of pcDNA-flag-HuR. To normalize the transfection efficiency, cells were cotransfected with 50 ng of pBind containing renilla luciferase. After 24 h, cells were washed with PBS and lysed using passive lysis buffer. Luciferase activity was measured using the Dual-Luciferase Reporter Assay kit (Promega GmbH) and a Wallac Victor 1420 Multilabel Counter (PerkinElmer, Waltham, MA, USA), according to the manufacturer’s instructions.

### Quantitative reverse transcription (qRT)-PCR

RNA was isolated using TRIzol reagent (Invitrogen Life Technologies, Carlsbad, CA, USA) according to the manufacturer’s instructions. First-strand cDNA synthesis was conducted using the iScript RT kit (Bio-Rad Laboratories, Hercules, CA, USA) in 20 *μ*l reaction solutions. Real-time PCR was conducted with the iQ™ SYBR^®^ Green Supermix (Bio-Rad Laboratories) in 20 *μ*l reaction solutions using the iCycler thermal cycler (Bio-Rad Laboratories). The relative RNA amount was calculated using the ΔΔCt method and normalized using glyceraldehyde-3-phosphate dehydrogenase (GAPDH) as an internal control.

### Actinomycin D (ActD) experiments

MDA-MB-231 cells were seeded into 6-well plates and transfected with the HuR overexpression vector (pcDNA-flag-HuR) using Lipofectamine™ 2000 (Invitrogen Life Technologies) according to the manufacturer’s instructions. After 48 h, MDA-MB-231 cells were treated with 5 *μ*g/ml transcription inhibitor ActD (Sigma, St. Louis, MO, USA). Total RNA was isolated at time intervals of 0, 2, 4 and 6 h following ActD addition. FOXO1 mRNA was determined using qRT-PCR, and the relative amount of FOXO1 mRNA without Act D treatment was set to 100%.

### Western blot analysis

MDA-MB-231 cells with indicated treatment were harvested and lysed in ice-cold radioimmunoprecipitation assay (RIPA) buffer consisting of 1% nonidet P-40, 0.1% SDS, 0.5% deoxycholate, 150 mM NaCl, 50 mM NaF, 1 mM DTT, 50 mM Tris-HCl, (pH 8.0) and a freshly prepared protease inhibitor mixture (Complete, Mini; Roche Applied Science, Burgess Hill, UK). Total protein concentration of lysates was determined using the Bio-Rad protein assay. A total of 40 *μ*g of protein lysates were separated using 12% SDS-PAGE and transferred onto nitrocellulose membranes. For detection of HuR and FOXO1, a rabbit polyclonal anti-HuR antibody (1:2000; Santa Cruz Biotechnology, Inc., Santa Cruz, CA, USA) or a rabbit monoclonal anti-FOXO1 antibody (1:3000; Abcam, Cambridge, UK) were used. The immuno-reactive proteins on the western blots were visualized using the enhanced chemiluminescence (ECL) detection system (Amersham Pharmacia Biotech, Amersham, UK).

### Immunoprecipitation qRT-PCR assay

MDA-MB-231 cells were seeded in 100 mm dishes, and after 24 h, 1% formaldehyde was added to the medium to crosslink protein-RNA. Cells were lysed in a buffer containing 10 mM HEPES (pH 7.9), 1.5 mM MgCl_2_, 10 mM KCl, 0.5 mM DTT, 0.1% NP-40, 50 mM NaF, 10 mM Na_3_VO_4_, 10 mM sodium pyrophosphate, 50 mM disodium glycerol phosphate, 10 nM okadaic acid, 0.2% VRC, 100 U/ml RNasin and 1/25 v/v complete EDTA-free protease inhibitor cocktail. The lysed cells were centrifuged at 12,000 × g for 10 min at 4°C and the supernatants were incubated with 30 *μ*g of unrelated antibody (IgG; Sigma) or anti-HuR at 4°C for 60 min. Once incubation was complete, agarose beads and 50 *μ*l of protein A/G were added and cells were incubated for a further 60 min at 4°C. Subsequently, the precipitated beads were washed with lysis buffer three times. The RNA in the immunoprecipitated complex and the RNA in the previously saved input fraction were released by incubating cells at 65°C for 2 h with 200 mM NaCl and 20 *μ*g proteinase K, which reversed the cross-linking. The RNAs were extracted as previously described. The amount of FOXO1 mRNA bound by HuR was determined by RT-PCR using the following primers: sense, 5′-TTGTTACATAGTCAGCTTG-3′; and antisense, 5′-TCACTTTCCTGCCCAACCAG-3′. PCR conditions were as follows: 95°C for 5 min, followed by 25 cycles of 95°C for 15 sec, 55°C for 20 sec and 72°C for 1 min.

### Caspase activity assay

MDA-MB-231 cells were seeded into 96-well plates at a density of 5×10^3^ cells/well for 24 h. Cells were transfected with control siRNA (siRNA-Con), HuR siRNA (siRNA-HuR) and a combination of siRNA-HuR and pcDNA-flag-FOXO1 respectively for 24 h, and treated with 5 *μ*g/ml 5-FU. After 24 h, 50 *μ*l of Caspase-Glo^®^ 3/7 Reagent (Promega GmbH) was added into each well and incubated for 1 h. The luminescence of each well was measured using the GENios Pro Multifunctional Reader (Tecan, Mannedorf, Switzerland).

### Annexin V staining

MDA-MB-231 cells were treated as previously described. After 24 h of 5 *μ*g/ml 5-FU treatment, MDA-MB-231 cells were washed three times with cold PBS. Cells were then incubated with 100 *μ*l binding buffer containing 2 *μ*g/ml Annexin V-fluorescein isothiocyanate (FITC; Roche Applied Science) and 10 *μ*g/ml of the vital dye propidium iodide for 10 min in the dark. Following further washes in PBS, the cells were analyzed using flow cytometry (BD Pharmingen, San Diego, CA, USA).

## Results

### HuR interacts with FOXO1 3′ UTR

**Figure 1.**
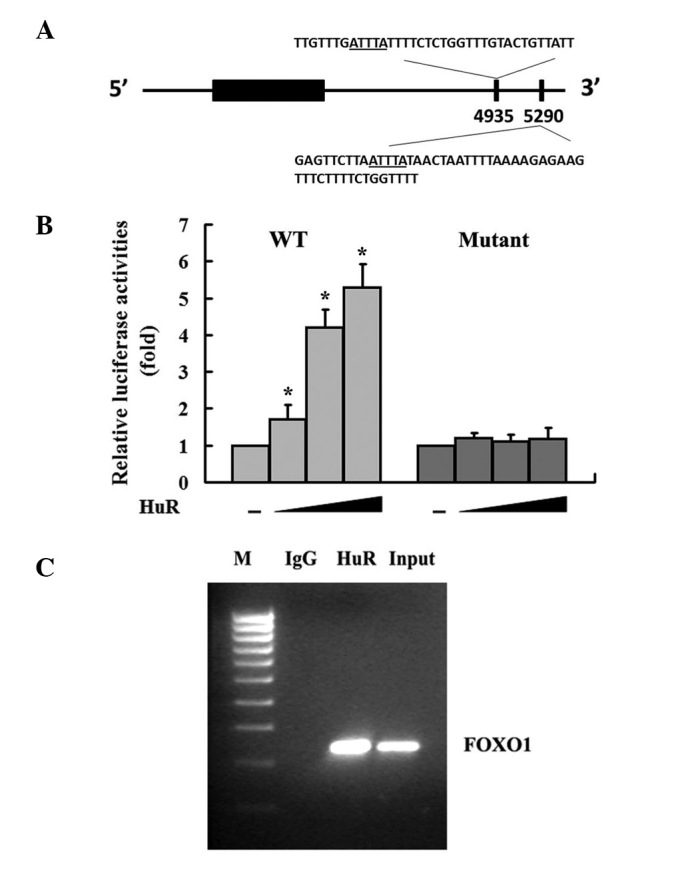
HuR upregulates FOXO1 expression. (A) Schematic representation of the FOXO1 mRNA 3′ UTR sequence. The HuR binding sequence ATTTA is underlined. (B) MDA-MB-231 cells were seeded in 24-well plates and transfected with 400 ng of WT or Mutant FOXO1 3′ UTRs in combination with increased doses of pcDNA-flag-HuR (100, 200 and 400 ng; n≥3). Activity of the firefly luciferase was normalized to that of the renilla luciferase. Values are expressed as means ± SEM of at least three independent experiments. ^*^P<0.05. (C) MDA-MB-231 cells were seeded in 100 mm dishes. After 24 h, cells were lysed and incubated with either anti-HuR or a nonspecific IgG antibodies and Protein A Sepharose^®^. Cytoplasmic extract not incubated with an antibody was saved as an ‘input’ sample. The immunoprecipitated RNAs were isolated and FOXO1 mRNA was amplified using RT-PCR. A nonspecific antibody was used as a negative control (IgG). Result presented is a representative of three different experiments. HuR, human ELAV/Hu protein; WT, wild type; M, DNA marker; FOXO1, Forkhead box protein O 1; UTR, untranslated region; RT-PCR, reverse transcription-PCR.

Bioinformatics analysis of the human FOXO1 mRNA revealed that there are two potential AU-rich elements (AREs) within the 3′ UTR (Fig. 1A); however, its role in the regulation of FOXO1 gene expression has not been elucidated. To address this, luciferase report constructs containing full length FOXO1 3′ UTRs (WT) or deletions of the AU-rich regions (Mutant) were utilized. Overexpression of HuR in MDA-MB-231 breast cancer cell lines caused a dose-dependent increase of luciferase activities (Fig. 1B). However, interference of HuR-FOXO1 interactions by overexpressing mutant FOXO1 3′ UTR abrogated the effect of HuR on FOXO1 3′ UTR (Fig. 1B). Taken together, these results suggest that the AREs within the 3′ UTR of FOXO1 are responsible for HuR-mediated upregulation of FOXO1. To further determine if HuR was directly associated with FOXO1 3′ UTR, we conducted an immunoprecipitation RT-PCR assay with primers that target the FOXO1 coding region. Using HuR antibodies, we were able to coimmunoprecipitate FOXO1 mRNA from MDA-MB-231 cells (Fig. 1C). No FOXO1-specific PCR product was identified when cell lysates were precipitated with IgG antibodies (Fig. 1C). These findings provide strong evidence that HuR binds specifically to FOXO1 mRNA in MDA-MB-231 cells *in vivo*.

### HuR overexpression stabilizes FOXO1 mRNA

**Figure 2.**
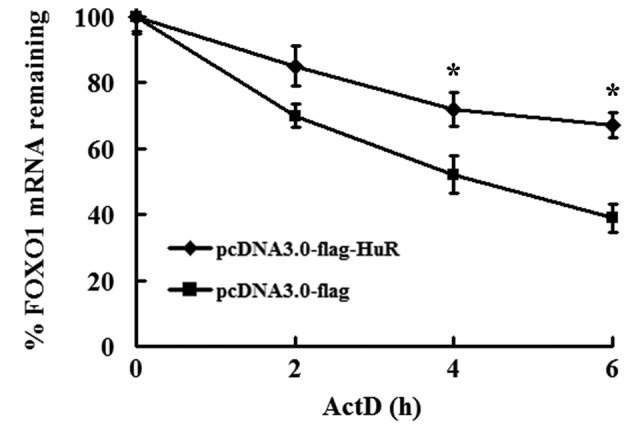
HuR overexpression stabilizes FOXO1 mRNA. MDA-MB-231 cells were seeded into 6-well plates and transfected with pcDNA-flag and pcDNA-flag-HuR plasmids using Lipofectamine™2000. After 48 h, cells were treated with 5 *μ*g/ml of the transcription inhibitor ActD. At the indicated time points, RNAs were isolated and FOXO1 mRNA was determined by RT-PCR. The relative amount of FOXO1 mRNA without ActD treatment was set to 100%. Values were expressed as means ± SEM of at least three independent experiments. ^*^P<0.05. FOXO1, Forkhead box protein O 1; ActD, actinomycin D. HuR, human ELAV/Hu protein; RT-PCR, reverse transcription-PCR.

Accumulating evidence indicates that HuR controls mRNA activity by regulating mRNA stability and/or translation (4,5). We revealed that HuR is involved in FOXO1 mRNA turnover. To exclude the influence of transcription, the transcription inhibitor ActD was used. As shown in Fig. 2, the half-life of FOXO1 mRNA in pcDNA3.0-flag-transfected cells was 4.7±0.3 h, while the half-life of FOXO1 mRNA in pcDNA3-HuR-flag-transfected cells was much longer (10.8±0.4 h) compared with the control vector (Fig. 2). These results clearly demonstrate that HuR stabilizes FOXO1 mRNA, which plays an important role in the regulation of FOXO1 gene expression.

### HuR positively regulates FOXO1 expression

**Figure 3.**
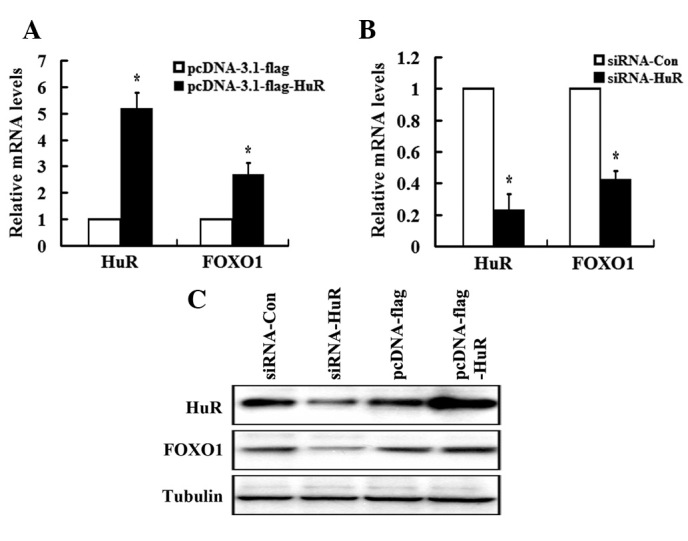
HuR positively regulates FOXO1 expression. MDA-MB-231 cells were seeded into 6-well plates and transfected with pcDNA-flag or pcDNA-flag-HuR plasmids and siRNA-Con or siRNA-HuR plasmids. (A and B) mRNA and (C) protein levels were determined by RT-PCR and western blot analysis, respectively. Tubulin was used as an internal control, and values are expressed as means ± SEM of at least three independent experiments. ^*^P<0.05. HuR, human ELAV/Hu protein; FOXO1, Forkhead box protein O 1; Con, control. RT-PCR, reverse transcription-PCR.

Considering the direct association of HuR and FOXO1 3′ UTR, combined with the increased stability of FOXO1 mRNA, we revealed that HuR positively regulates FOXO1 expression. To study this, the loss- and gain-of-function of HuR approach was utilized. HuR expression was confirmed upon transient transfection with pcDNA3.0-flag or pcDNA3-HuR-flag plasmids (Fig. 3A) and a scrambled siRNA or a specific siRNA plasmid for HuR (Fig. 3B and C) by qRT-PCR and western blot analysis. Fig. 3B shows that FOXO1 mRNA levels were reduced by approximately 60% in siRNA-HuR transfectants relative to the levels of FOXO1 mRNA in cells transfected with a scrambled siRNA. In accordance with this, overexpression of HuR resulted in a 2.8- and 2.1-fold increase of FOXO1 expression at mRNA and protein levels, respectively (Fig. 3A and C).

### 5-FU induces FOXO1 expression in a HuR-dependent manner

**Figure 4.**
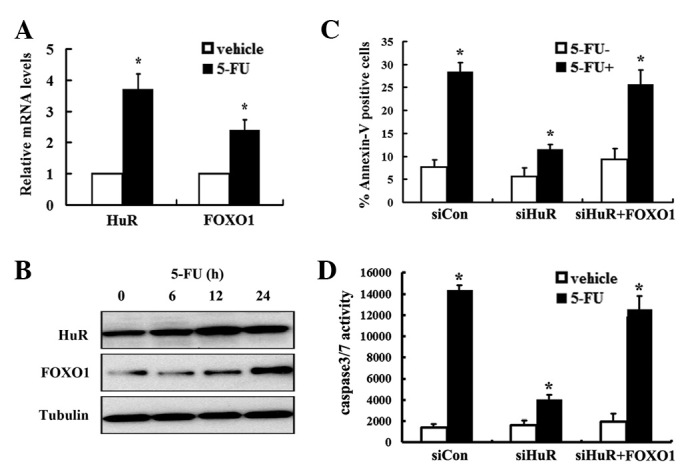
5-FU induces FOXO1 expression in a HuR-dependent manner. MDA-MB-231 cells were seeded into 6-well plates and treated with 5 *μ*g/ml 5-FU. At the indicated time, cells were collected and (A) RT-PCR and (B) western blot analysis were conducted to detect HuR and FOXO1 expression. (C) MDA-MB-231 cells were seeded into 6-well plates and transfected with siRNA-Con, siRNA-HuR and FOXO1 overexpression and control lentivirus, respectively. After 24 h, cells were treated with 5-FU. Following 24 h of incubation, the apoptotic cells were measured by PI and Annexin V-FITC staining and analyzed by flow cytometry. Values are expressed as mean ± SEM of at least three independent experiments. ^*^P<0.05. (D) Cells were treated as described above. Caspase-3 activities were measured using a Caspase-Glo 3/7 assay kit. Values are expressed as mean ± SEM of at least three independent experiments. ^*^Indicates P<0.05. 5-FU, 5-fluorouracil; HuR, human ELAV/Hu protein; FOXO1, Forkhead box protein O 1; Con, control. RT-PCR, reverse transcription-PCR; PI, propidium iodide; FITC, fluorescein isothiocyanate.

Previous studies demonstrate that HuR increases mRNA stability of target genes upon stimuli. We demonstrated that HuR regulated FOXO1 expression upon 5-FU treatment in MDA-MB-231 cells. Notably, 5-FU-induced accumulation of HuR occurred in correlation with an increase in the FOXO1 mRNA level, which exhibited a dose- and time-dependent manner (Fig. 4A and B). To investigate the functional role of HuR in the regulation of FOXO1 expression and control of apoptosis in MDA-MB-231 cells upon 5-FU treatment, Annexin V assays were conducted and caspase-3 activity was determined. qRT-PCR analysis revealed that HuR mRNA was reduced in HuR siRNA-transfected cells to ∼30% of the level found in cells transfected with a control siRNA (data not shown). 5-FU increased the level of Annexin V positive cells by 3.8-fold compared with the vehicle control (Fig. 4C); however, 5-FU-induced apoptosis was significantly inhibited by HuR knockdown (Fig. 4C). Consistently, 5-FU induced a 10.3-fold increase of caspase-3 activity compared with vehicle treatment (Fig. 4D). Conversely, 5-FU treatment resulted in a reduced increase of caspase-3 activity upon silencing HuR (Fig. 4D), which indicates that HuR knockdown remarkably abrogates 5-FU-induced apoptosis. To determine whether FOXO1 is involved in HuR-mediated apoptosis upon 5-FU treatment, FOXO1 overexpression lentivirus was utilized. FOXO1 mRNA and protein levels were significantly enhanced upon transduction of FOXO1 overexpression lentivirus (data not shown). As expected, overexpression of FOXO1 restores 5-FU-induced apoptosis upon HuR knockdown determined by Annexin V and caspase-3 activity assays (Fig. 4C and D). Taken together, our data suggest that HuR-mediated regulation of FOXO1 plays a critical role in 5-FU-induced apoptosis.

## Discussion

As a critical transcription factor, FOXO1 orchestratedly regulated genes involved in cell cycle inhibition (e.g., p27), apoptosis (e.g. Bim, FASL and TATRAIL) and DNA repair (e.g., GADD45a) under stress or differentiation conditions (16–20). The majority of previous studies with regard to FOXO1 function were closely associated with its phosphorylation and acetylation modification (21,22). A number of studies conclusively demonstrate that the regulation of FOXO1 expression by growth factors and other stimuli occurs predominantly at the post-translational level. However, there is little understanding of the specific RNA-protein interactions involved in the regulation of FOXO1 expression and function. We postulate that post-transcriptional regulation, which controls the mRNA level of FOXO1, is also critical for its function. A new finding recently disclosed in endometrial cancer cell lines suggested that the lack of FOXO1 expression was associated with an increased mRNA turnover with an unknown mechanism (23). Our study revealed that HuR enhanced the expression of FOXO1 at the post-transcriptional level by increasing FOXO1 mRNA stability (Fig. 2), adding a novel molecular mechanism of how FOXO1 is regulated in addition to translational regulation.

FOXO1 is the most abundant FOXO isoform in insulin-responsive tissues including hepatic, adipose and pancreatic cells (21,22). However, as a critical tumor suppressor, FOXO1 expression has been observed to be undetectable or extremely low in certain tissues, including prostate, breast and colon cancer cells (24–26), which suggests that low levels of FOXO1 may be one of the factors contributing to the oncogenesis and progression of breast carcinoma. Our study provides the first evidence that 5-FU treatment enhances FOXO1 expression by stabilizing its mRNA level via HuR. This suggests that modulating FOXO1 expression may serve as a novel strategy to sensitize breast cancers to chemotherapy and/or radiotherapy.
